# Monitoring the electron dynamics of the excited state via higher-order spectral minimum

**DOI:** 10.1038/s41598-017-10667-6

**Published:** 2017-09-04

**Authors:** Cai-Ping Zhang, Chang-Long Xia, Xiang-Fu Jia, Xiang-Yang Miao

**Affiliations:** 1 College of Physics and Information Engineering, Shanxi Normal University, Linfen, 041004 China; 2 College of Chemistry and Materials Science, Shanxi Normal University, Linfen, 041004 China

## Abstract

A pump-probe scheme for monitoring the electron dynamics of the excited state has been investigated by numerically solving the two-state time-dependent Schrödinger equation based on the non-Born-Oppenheimer approximation. By adjusting the delay time between a mid-infrared probe pulse and an ultra violet pump pulse, an obvious minimum can be seen in the higher-order harmonic region. With electron probability density distribution, ionization rate and classical simulation, the minimum can be ascribed to the electron localization around one nucleus at larger delay time and represents the electron dynamics of the excited state at the time of ionization. Moreover, the position of the minimum is much more sensitive to the nuclear motion.

## Introduction

To trace the dynamic processes in atoms and molecules, researchers have made big process in the development of the ultrashort laser pulse^1^. Nowadays the pulse with duration of 67 as has been achieved experimentally^2^, which makes it possible to probe, manipulate and control the electron dynamics in photochemistry and attosecond physics^3^. Based on the pump-probe scheme^4, 5^, Bandrauk *et al*. have observed the attosecond electron motion between coherent electronic states by measuring the photoelectron signal and the photoelectron angular distribution as function of the pump-probe delay time (*t*_*del*_)^6, 7^. In this scheme, the duration of the probe pulse is shorter than the electron oscillatory period. Moreover, it’s known that the high-order harmonic generation (HHG) can be well explained by three-step model^8^: firstly, the electron tunnels through the potential barrier, and then is accelerated in the external field; finally, when the laser field changes its direction, the electron may return and recombine with the parent ion with the emission of harmonic photons. The highest harmonic energy is *I*_*p*_ + 3.17 *U*_*p*_, where *I*_*p*_ is the ionization potential and ${U}_{p}=\frac{{E}^{2}}{4{\omega }^{2}}$ is the ponderomotive energy with *E* and *ω* being the peak amplitude and the laser frequency, respectively. So each step in HHG process occurs on the femtosecond or attosecond time scale, in other words, the electron dynamics can also be traced by the high time-revolution HHG spectrum^8, 9^. Moreover, many interesting physical phenomena have been found with the infrared or mid-infrared probe pulse. Wang *et al*. have shown that the minimum electron probability density distribution of Rydberg atom corresponds to the dip-node structure on the harmonic spectrum^10, 11^. Wörner *et al*. have reported experimentally that both the harmonic amplitude and phase can detect the chemical reaction based on the harmonic interference between the excited and ground states^12, 13^. Bandrauk *et al*. have monitored the coherent attosecond dynamics and manipulated the interferences by adjusting the population of electronic states and the *t*_*del*_^14, 15^. Kraus *et al*. have provided the experimental demonstration about measuring an electronic coherence through HHG^16^.

In previous studies, the lower-order harmonics have been widely used to detect the electron dynamics of the excited state^17–19^. It’s found that the excited state manifests the dynamics in the hollow region originating from the two-center interference^17^ or the resonance region between the ground state and the excite state^18, 19^. How do the higher-order harmonics code the electron dynamics of the excited state, which play a key role in the generation of ultrashort pulse^1^? Considering the fast dissociation of ${{\rm{H}}}_{2}^{+}$ at excited state blurring some dynamic information, we have adopted the pump-probe scheme to prepare ${{\rm{T}}}_{2}^{+}$ in the superposition state of the ground and excited states to further detect the electron dynamics of the excited state with the higher-order harmonics by solving the two-state time-dependent Schrödinger equation (TDSE) based on the non-Born-Oppenheimer (NBO) approximation. By adjusting the *t*_*del*_, the spectral minimum can be observed at large *t*_*del*_ in the higher-order harmonic region, which can be viewed as a signature of the electron dynamics of the excited state. The time-frequency distribution, classical kinetic energy map and ionization rate distribution are adopted to reveal the underlying physical mechanism.

## Results

**Figure 1:**
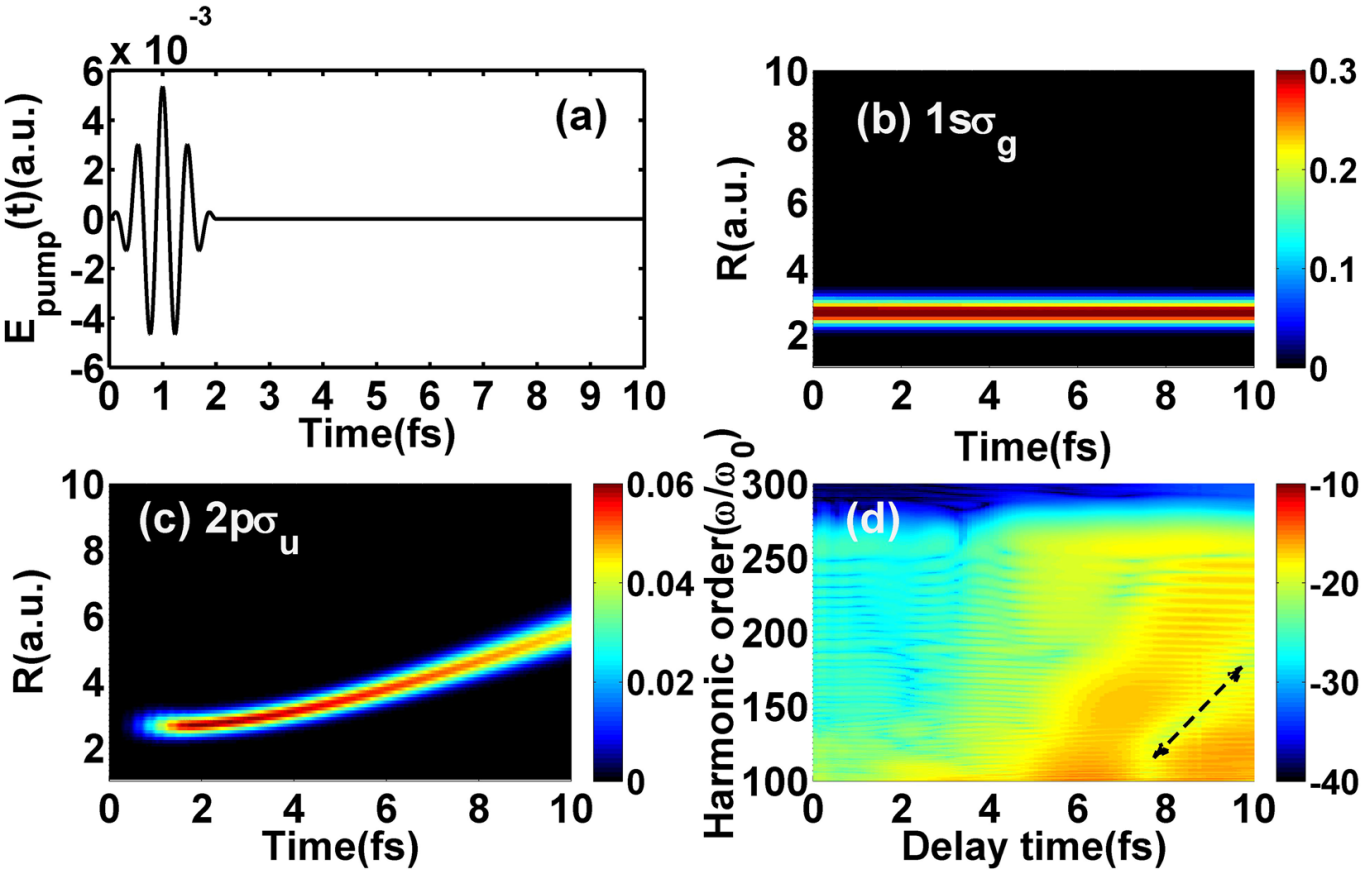
(**a**) Sketch of the electric field of the pump pulse. (**b**,**c**) The nuclear probability density distribution of ${{\rm{T}}}_{2}^{+}$ at 1s*σ*_*g*_ (2p*σ*_*u*_) state in the pump field. (**d**) Dependence of harmonic spectra on *t*_*del*_.

The model of ${{\rm{T}}}_{2}^{+}$ is initially in the electronic and vibrational ground state, and the corresponding *I*_*p*_ of the ground state (1s*σ*_*g*_) is 31.1 eV at *R* = 2.6 a.u. (the equilibrium separation). We first adopt an ultra violet pump pulse with the wavelength of 148 nm to excite part of wave packets into the repulsive first excited state (2p*σ*_*u*_) via one-photon resonance. The electric field of the pump pulse $E(t)=-\frac{1}{c}\frac{\partial }{\partial t}A(t)$ is displayed in Fig. 1(a). Here *A*(*t*) is the vector potential with sine-squared envelope $A(t)=-\frac{c}{\omega }{E}_{0}{\rm{si}}{{\rm{n}}}^{2}(\pi t/{t}_{tot}){\rm{\sin }}(\omega (t-{t}_{tot}\mathrm{/2))}$. The total duration (*t*_*tot*_) and the peak intensity are 2 fs and 1.0 × 10^12^ W/cm^2^, respectively. And then the molecular ion will be prepared on a coherent superposition of 1s*σ*_*g*_ state and 2p*σ*_*u*_ state. Moreover, the wave packets at 1s*σ*_*g*_ state mainly distribute around the equilibrium distance as shown in Fig. 1(b) while those at 2p*σ*_*u*_ state propagate to larger internuclear distance along the potential curve as shown in Fig. 1(c). At end of the pump pulse, the total excitation probability (i.e. population of the 2p*σ*_*u*_ state) is 2.7%. After a *t*_*del*_, the initial coherent superposition wave packets are evolved by a mid-infrared probe pulse with identical vector potential to the pump pulse to generate intense harmonics. Here, the *t*_*del*_ is defined as the time difference between the ending of the pump pulse and the beginning of the probe pulse. The wavelength, total duration and peak intensity of the probe pulse are 1600 nm, 10 fs and 3.0 × 10^14^ W/cm^2^, respectively. Furthermore, the related harmonic spectra as function of *t*_*del*_ are plotted in Fig. 1(d). Interestingly, the harmonic intensities decay in the higher-order harmonic region at larger *t*_*del*_ (i.e. from *t*_*del*_ = 8 fs to *t*_*del*_ = 10 fs) as the dash arrow depicted. Moreover, the position of spectral minimum shifts as function of *t*_*del*_. In the following section, we will discuss these findings in detail.

## Discussion

With the initial coherent superposition of the 1s*σ*_*g*_ and 2p*σ*_*u*_ states, four channels can contribute to the HHG process. On the one hand, the electron can be ionized from and recombine to the 1s*σ*_*g*_ (2p*σ*_*u*_) state, i.e. H_11_ channel (H_22_ channel). On the other hand, it can also be ionized from the 1s*σ*_*g*_ (2p*σ*_*u*_) state and recombine to the 2p*σ*_*u*_ (1s*σ*_*g*_) state, i.e. H_12_ channel (H_21_ channel). As reported by Bredtmann *et al*. in previous studies^9, 14, 15^, two states tend to lose the nuclear overlap with the increase of the *t*_*del*_ so that only H11 channel and H22 channel contribute to the harmonic emission at larger *t*_*del*_. Moreover, we have simulated that the harmonic efficiency of the 1s*σ*_*g*_ state is over 6 orders of magnitude lower than that of the 2p*σ*_*u*_ state for *t*_*del*_ = 0 fs, and the difference of harmonic intensity can be further enhanced at larger *t*_*del*_. Accordingly, the excited state plays an important role in harmonic emission. Furthermore, it is the electron dynamics of the excited state that induces the spectral minima as the arrow illustrated in Fig. 1(d).

**Figure 2:**
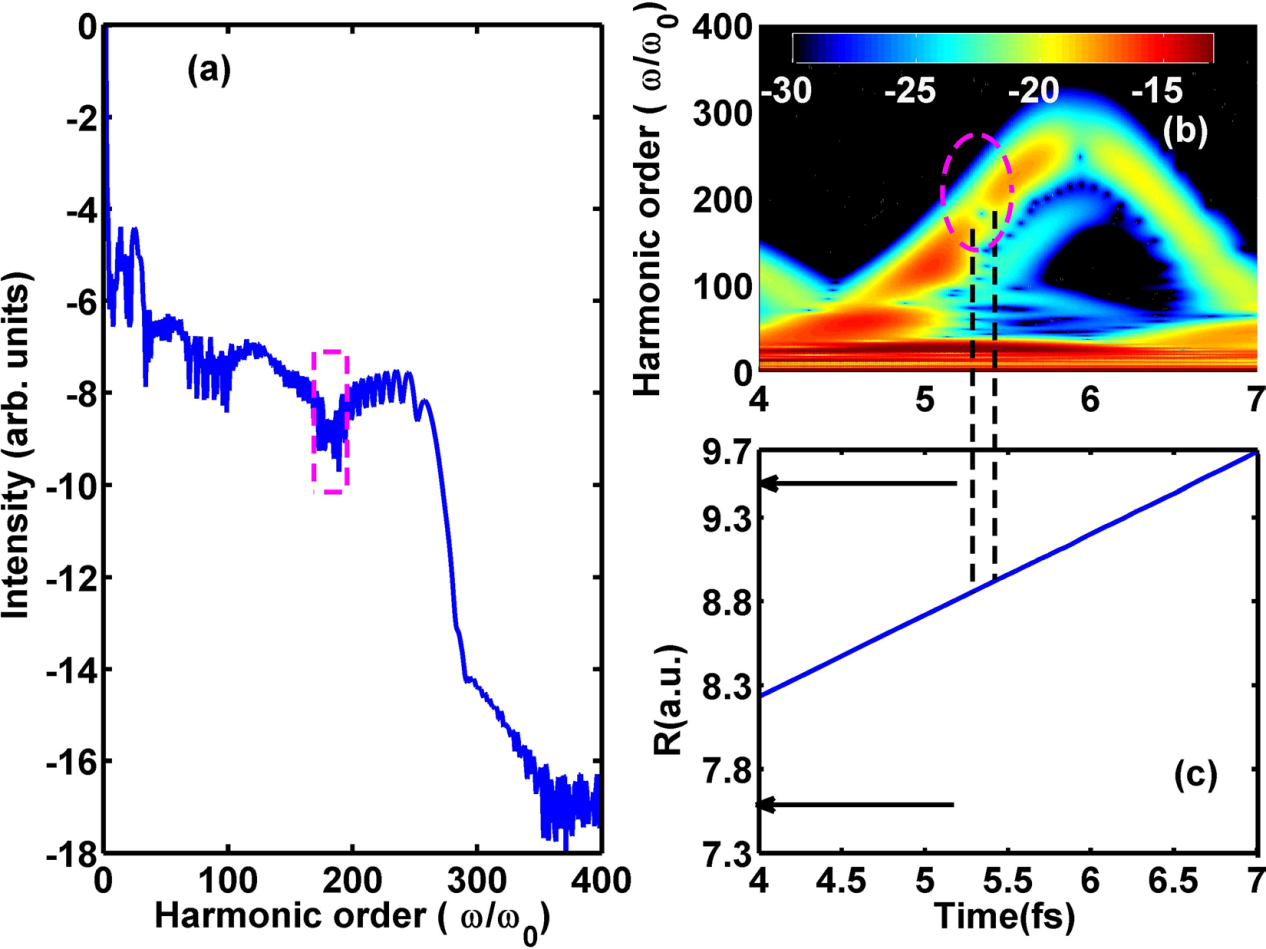
(**a**,**b**) are the harmonic spectrum and the time-frequency map at the superposition of the 1s*σ*_*g*_ and 2p*σ*_*u*_ states for *t*_*del*_ = 10 fs, respectively. (**c**) Related internuclear distance of ${{\rm{T}}}_{2}^{+}$ initially at 2p*σ*_*u*_ state.

To uncover the underlying mechanism, the harmonic spectrum from the superposition state for *t*_*del*_ = 10 fs has been shown in Fig. 2(a), which exhibits an obvious dip from 175th to 189th. The corresponding time-frequency map from 4 fs to 7 fs illustrates the electron dynamics in the probe field both in frequency and time dimensions in Fig. 2(b). It can be seen that the minimum noted by rectangle in harmonic spectrum corresponds to the weak harmonic emission from 5.2 fs to 5.4 fs noted by dash ellipse in time-frequency map. What is the source of the spectral minimum for single-electron system ${{\rm{T}}}_{2}^{+}$, the phase difference between the propagating and residual wave packets^12, 13^ or the two-center interference^17^? Since there is no longer nuclear overlap between the 1s*σ*_*g*_ state and the 2p*σ*_*u*_ state for *t*_*del*_ = 10 fs, the minimum is distinct from the dynamical minimum arising from the destructive phase difference with the returning wave packet in acceleration process. To check whether the minimum resulting from the two-center interference, the internuclear distance of ${{\rm{T}}}_{2}^{+}$ initially at 2p*σ*_*u*_ state calculated by $R(t)=\frac{\iint RdRdz{|\psi (R,z;t)|}^{2}}{\iint dRdz{|\psi (R,z;t)|}^{2}}$ has been displayed in Fig. 2(c) due to its dominated role in harmonic emission. For the 2p*σ*_*u*_ state with an antisymmetric orbital^17^, the two-center interference is governed by the term $I(k)={e}^{-ik\cdot R\mathrm{/2}}-{e}^{ik\cdot R\mathrm{/2}}=-2i\,\sin (k\cdot R\mathrm{/2)}$, where *I*(*k*) is the harmonic intensity, *k* is the electron momentum and *R* is the internuclear distance. The two-center interference minima are expected to occur when the argument of the sine is the integral multiple of 2*π*, i.e. *k* · *Rcosα* = 2*nπ* and ${N}_{min}\omega ={k}^{2}\mathrm{/2(}n=0,1,2\ldots ,)$, where *N*_*min*_ is the harmonic order at the two-center interference minimum and *ω* is the laser frequency of the probe pulse. For the two-center interference minimum from 175th to 189th at 2p*σ*_*u*_ state, the internuclear distance should satisfy with *R* = 1.91 n ~1.98 n (n is integer) at recombination moment. So the internuclear distance should be around 7.6 a.u. (n = 4) and 9.5 a.u. (n = 5) as arrows depicted in Fig. 2(c) during the time region from 5.2 fs to 5.4 fs. However, the related internuclear distance of ${{\rm{T}}}_{2}^{+}$ changes from 8.7 a.u. to 8.9 a.u. Consequently, the minimum cannot be ascribed to the two-center interference. As discussion above mentioned, the causative effect can not be found from the electron dynamics both in acceleration and recombination processes. How about the electron dynamics in ionization process?

**Figure 3:**
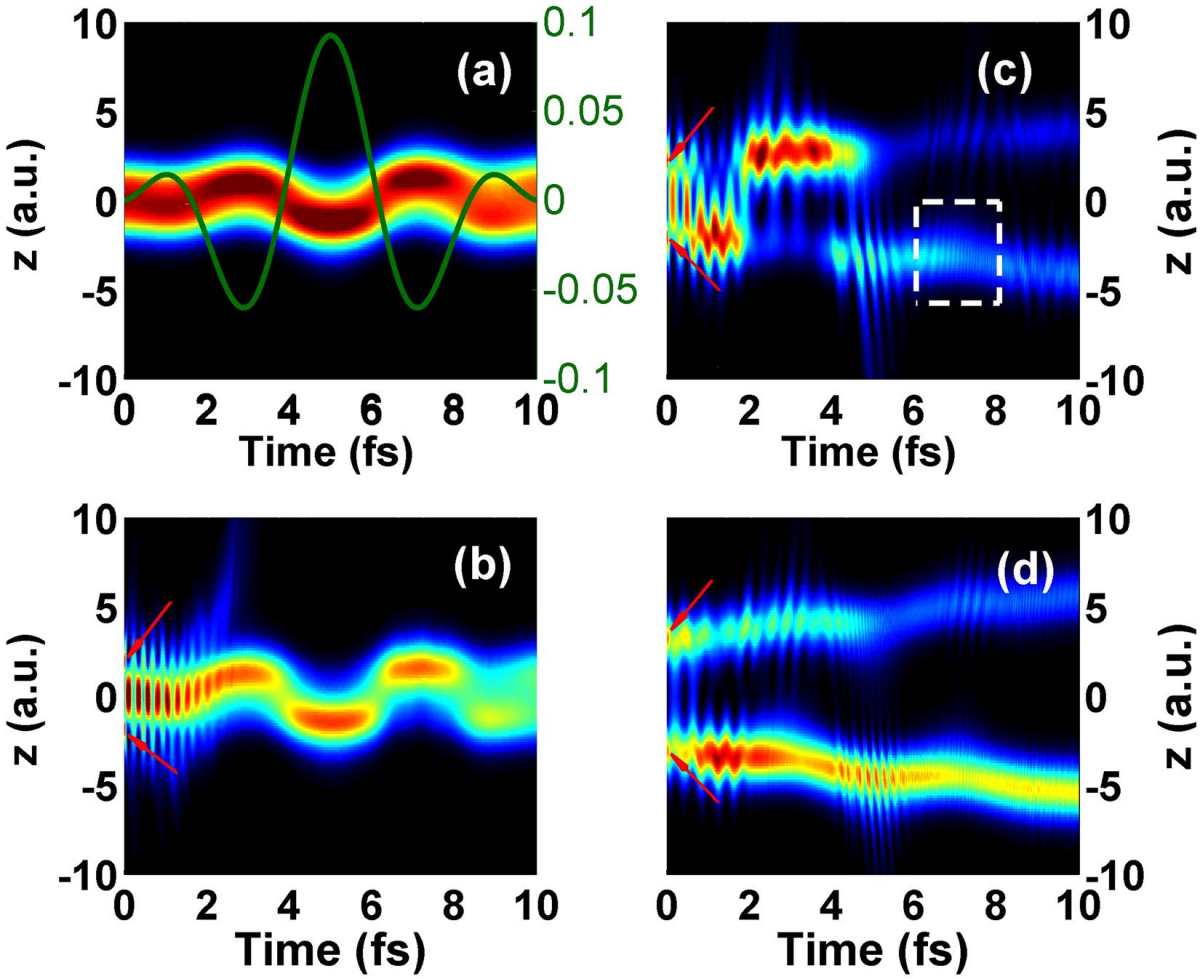
(**a**) The probability distribution of electron initially at 1s*σ*_*g*_ state in the probe field. The green line illustrates the electric field of the probe pulse. (**b**–**d**) The probability distributions of electron initially at 2p*σ*_*u*_ state in the probe field for *t*_*del*_ = 0 fs, *t*_*del*_ = 5 fs and *t*_*del*_ = 10 fs, respectively.

**Figure 4:**
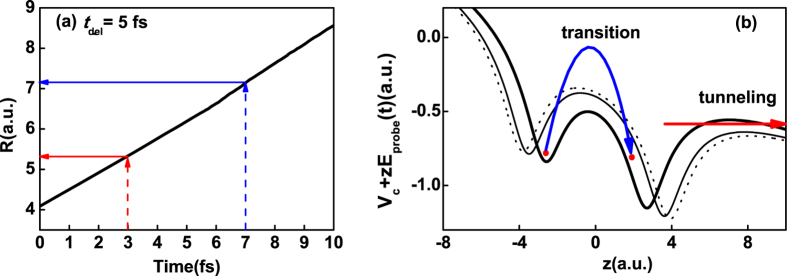
(**a**) The time-dependent internuclear distance of ${{\rm{T}}}_{2}^{+}$ in the probe field with initial 2p*σ*_*u*_ state for *t*_*del*_ = 5 fs. (**b**) The combined energy of the Coulomb potential and static electric field potential at *t* = 3 fs (thick line), *t* = 7 fs (thin line) for *t*_*del*_ = 5 fs and *t* = 3 fs (dotted line) for *t*_*del*_ = 10 fs, respectively. The dot represents the electron.

**Figure 5:**
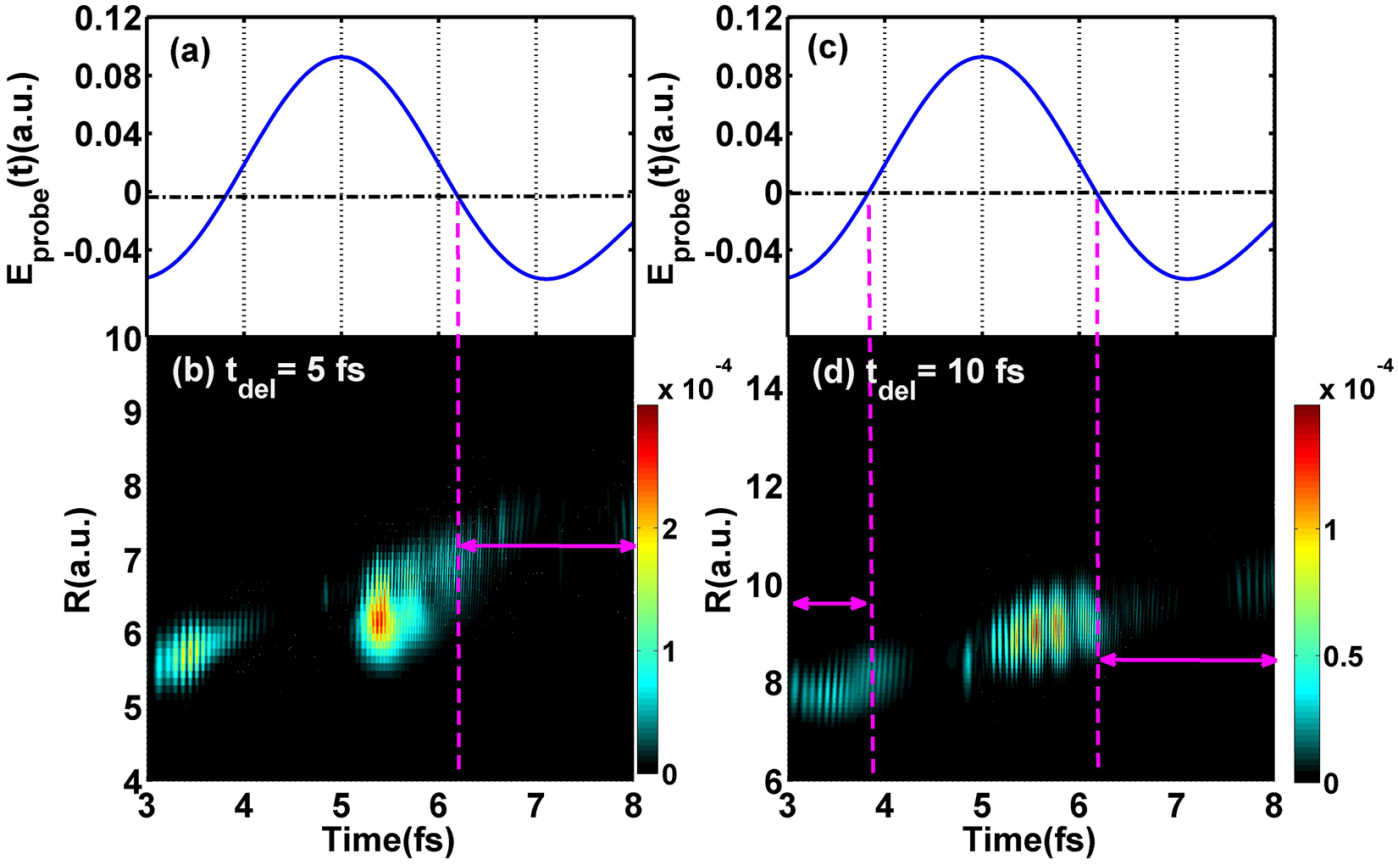
(**a**,**c**) The electric field of the probe pulse. (**b**,**d**) The related ionization rate distributions of ${{\rm{T}}}_{2}^{+}$ with initial 2p*σ*_*u*_ state for *t*_*del*_ = 5 fs and *t*_*del*_ = 10 fs, respectively.

To further discuss the effect of the ionization process on spectral minimum, we have calculated the probability distributions of the electron initially at the 1s*σ*_*g*_ and 2p*σ*_*u*_ states in the probe field by $P(z,t)={\int }_{1.0}^{26.65}{|\psi (R,z;t)|}^{2}dR$ in Fig. 3. Kamta *et al*. have revealed that in a strong field the *σ*_*u*_ electron always localizes in the direction of the field whereas the *σ*_*g*_ electron localizes opposite to the field^20^. This is due to the difference in polarizability that all ground states have negative polarizability and Stark shifts whereas excited states change sign^20^. As shown in Fig. 3(a), the electron initially at 1s*σ*_*g*_ state oscillates around two nuclei, which is opposite to the direction of the probe field (green line). However, the electron initially at 2p*σ*_*u*_ state presents different characters for different *t*_*del*_. The electron mainly distributes around *z* = 0 for *t*_*del*_ = 0 fs in Fig. 3(b) while with the increase of the *t*_*del*_, it tends to localize in the region of *z* < 0 obviously after *t* = 4 fs as shown in Fig. 3(c) and (d). Here *t* = 0 fs represents the beginning of the probe pulse. In the probe field, the electron initially at 2p*σ*_*u*_ state with antisymmetric distribution around *t* = 0 fs (as red arrow shown) may experience such physical processes: firstly, when the intensity is lower at the beginning of the probe pulse, the wave packet at 2p*σ*_*u*_ state will be transferred to the 1s*σ*_*g*_ state, and then spreads to the larger internuclear distance; finally, with the increase of the laser intensity, the wave packet experiences the complete ionization-acceleration-recombination process to generate efficiency harmonics. For *t*_*del*_ = 0 fs, the initial wave packet at 2p*σ*_*u*_ state localizing around 2.6 a.u. can be easily transfered to the 1s*σ*_*g*_ state between *t* = 0 fs to *t* = 2 fs, and then oscillates in the bottom of the potential cure of 1s*σ*_*g*_ state which induces the electron density distribution mainly around *z* = 0 as shown in Fig. 3(b). However, the spread of the wave packet will be more obvious in the probe field after the transfer process when the initial wave packet at 2p*σ*_*g*_ state distributes in larger internuclear distance for larger *t*_*del*_. For example, the electron density distributes prominently in the region of *z* < 0 after *t* = 4 fs for *t*_*del*_ = 5 fs as displayed in Fig. 3(c). What’s more, for *t*_*del*_ = 5 fs, the electron mainly localizes in the region of *z* < 0 as depicted in the rectangle around *t* = 7 fs whereas it should localize in the region of *z* > 0 as reported by Kamta *et al*.^20^. Moreover, this counter-intuitive phenomenon tends to appear at earlier time with the increase of *t*_*del*_ (e.g. around *t* = 3 fs for *t*_*del*_ = 10 fs shown in Fig. 3(d)). To get the abnormal phenomenon across, the electron motion in double-well potential at different times will be illustrated in Fig. 4. From the internuclear distance of ${{\rm{T}}}_{2}^{+}$ (black solid line) initially at 2p*σ*_*g*_ state for *t*_*del*_ = 5 fs in Fig. 4(a), the internuclear distances are 5.35 a.u. at *t* = 3 fs (as red arrow pointed) and 7.15 a.u. at *t* = 7 fs (as blue arrow pointed), respectively. And then the combined energies of the Coulomb potential and the static potential of 5.35 a.u.(thick line), 7.15 a.u. (thin line) for *t*_*del*_ = 5 fs and 7.75 a.u. at *t* = 3 fs (dotted line) for *t*_*del*_ = 10 fs are plotted in Fig. 4(b), respectively. With lower and narrower inner potential barrier under the condition of 5.35 a.u. at *t* = 3 fs, the electron can easily transit from the higher potential well to the lower one (as blue crooked arrow shown) and then tunnels though the potential well (as red horizontal arrow shown). However, the inner potential barrier becomes higher and wider with the nuclear separation. In this case, the transfer process will be suppressed, which probably induces the electron localization around the nucleus in the region of *z* < 0 around *t* = 7 fs for *t*_*del*_ = 5 fs. And then the related ionization process will also be suppressed. So does for the case of *t*_*del*_ = 10 fs. Moreover, the ionization rate distributions *P*_*ion*_(*R*, *t*) for *t*_*del*_ = 5 fs and *t*_*del*_ = 10 fs have also been provided in Fig. 5(c) and (d), which can be obtained based on the flux operator ${P}_{ion}(R,t)=IM[\langle \psi (R,z;t)|\delta (z-{z}_{s})\frac{\partial }{\partial z}|\psi (R,z;t)\rangle ]$, where *z*_*s*_ = 40 a.u. represents the position for flux analysis. Obviously, the ionization rate is much lower when the electron distributes in the region of *z* < 0 in negative probe field as the double-arrow shown, which further verifies the electron dynamics above analyzed. In general, the electron localization will be abnormal when the internuclear distance is larger than 6.7 a.u. (i.e. *t* > 6.1 fs for *t*_*del*_ = 5 fs). Moreover, it agrees well with the result reported by Fujimura *et al*.^21^ that the electron distribution is counter-intuitive in the coupling region of 1s*σ*_*g*_ and 2p*σ*_*u*_ states. Therefore, the minimum is probably ascribed to the suppression of the ionization process when the potential curves of two lowest electronic states couple together. Additionally, the wave packet tends to spread to 6.8 a.u. around *t* = 3 fs in the probe field for *t*_*del*_ = 8 fs so that the electron localization in the region of *z* < 0 would lead to the ionization suppression. Thus the obvious spectral minimum can be clearly seen for *t*_*del*_ > 8 fs in Fig. 1(d). Next, we will further explore the mechanism of position shifting as function of *t*_*del*_ shown in Fig. 1(d).

**Figure 6:**
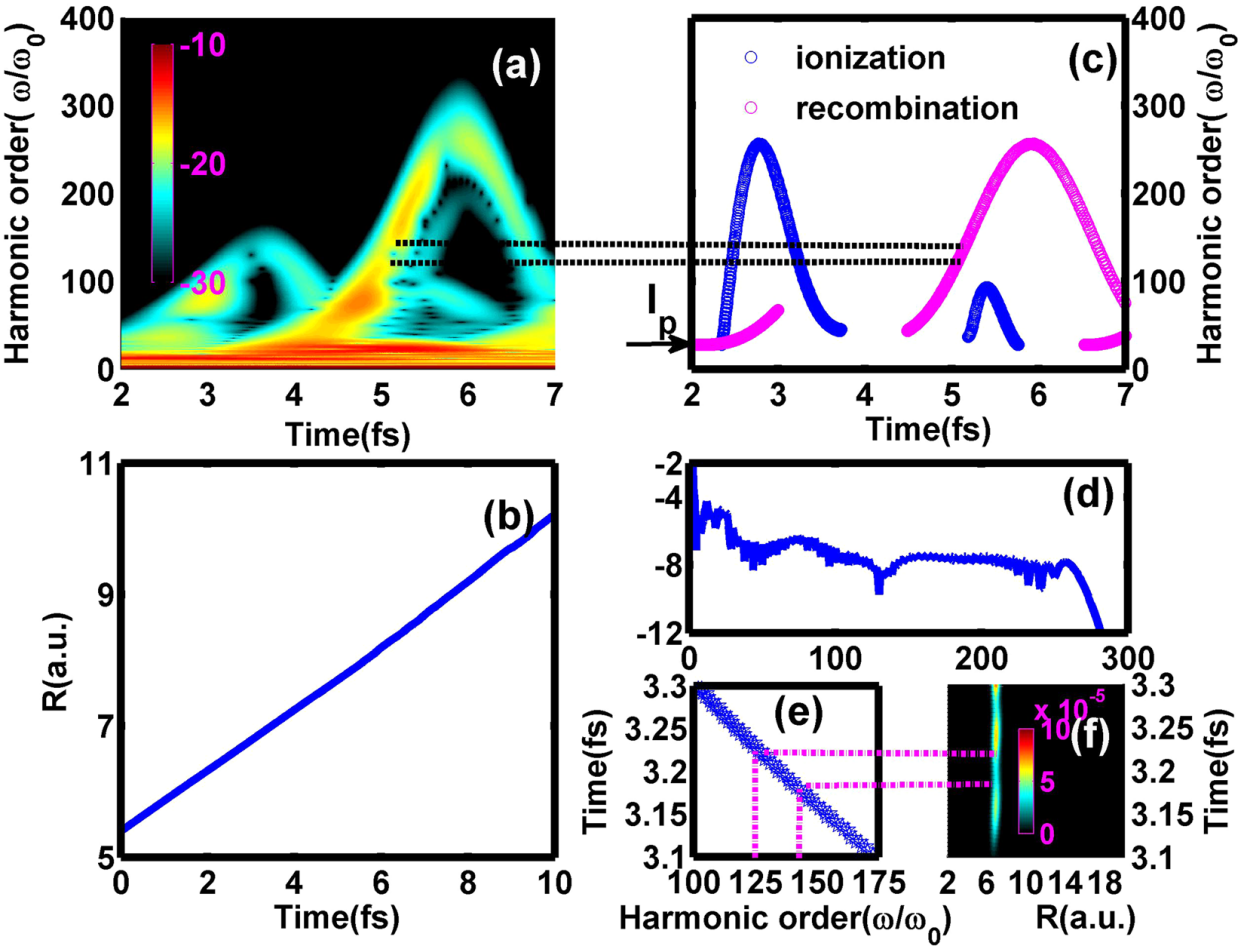
(**a**) The time-frequency map of ${{\rm{T}}}_{2}^{+}$ with initial superposition state for *t*_*del*_ = 8 fs. (**b**) The time-dependent internuclear distance of ${{\rm{T}}}_{2}^{+}$ with initial 2p*σ*_*u*_ state in the probe field. (**c**) and (**d**) are the classical kinetic energy map and related harmonic spectrum, respectively. (**e**) The enlargement of (**c**) from 3.1 fs to 3.3 fs. (**f**) The corresponding ionization rate distribution.

Figure 6(a) illustrates the time-frequency map of ${{\rm{T}}}_{2}^{+}$ from *t* = 2 fs to *t* = 7 fs for *t*_*del*_ = 8 fs. In this condition, the spectral minimum distributes in lower energy region from 125th to 141th (as black dash line shown) compared with the case of *t*_*del*_ = 10 fs shown in Fig. 2(b). As the spectral minimum origins from the ionization suppression, the classical simulation by solving the Newton’s equations of motion^22, 23^ can be used to judge the ionization moments for different *t*_*del*_. Seen from Fig. 6(b), the internuclear distance changes far less than $\frac{E}{{\omega }^{2}}$ for *t*_*del*_ = 8 fs, so similar cutoff energies can be obtained both from the recombinations with parent nucleus and neighboring nucleus^22^. Here we just provide the classical simulation about the former case, moreover, the multiple rescattering events have also been neglected considering the appearance of the spectral minimum in first rescattering in quantum simulation. According to the discussion about the electron motion above mentioned, the ionization process of ${{\rm{T}}}_{2}^{+}$ mainly takes place around *t* = 3 fs in the probe field when the internuclear distance is about 6.8 a.u. for *t*_*del*_ = 8 fs, and the related *I*_*p*_ = 0.79 a.u. corresponds to 27.7th harmonic order. The cutoff energy (about 284.7th harmonic order) based on the classical simulation agrees well with the quantum ones shown in Fig. 6(a) and (d). Furthermore, due to the critical role of the kinetic energy (about 257th harmonic order) obtained in the probe field, the cutoff energy is almost unchanged with the increase of *t*_*del*_ as displayed in Fig. 1(d). As seen from the classical simulation from 3.1 fs to 3.3 fs depicted in Fig. 6(e), it is the ionization process from 3.18 fs to 3.23 fs (as magenta dash line shown) that contributes to the spectral minimum from 125th to 141th. Moreover, it corresponds to the lowest ionization rate for *t*_*del*_ = 8 fs shown in Fig. 6(f). With the identical method, the lowest ionization rate for *t*_*del*_ = 10 fs will shift to earlier moments from 3.06 fs to 3.1 fs, which means that the spectral minimum will exist in higher energy region with the increase of *t*_*del*_ as shown in Fig. 1(d). In other words, the position of the spectral minimum depends on the nuclear motion.

In conclusion, the electron dynamics of the excited state has been detected with the higher-order spectral minimum by solving the two-state TDSE. With the increase of *t*_*del*_ between the pump and probe pulses, the excited state tends to dominate the harmonic emission. Moreover, the electron localization around one nucleus in the coupling region of both electronic states would result in the suppression of ionization process. As a consequence, the spectral minimum can be observed in higher-order harmonic region with interesting characters: (i) the minimum appears at certain photon energy region; (ii) the position of the minimum shifts as function of *t*_*del*_. In general, this work will provide new insight into the electron dynamics of excited state in strong-field physics. Furthermore, with the close relationship to the nuclear motion, the spectral minimum may be used to probe the nuclear dynamics in turn.

## Methods

The one-dimensional calculation of ${{\rm{T}}}_{2}^{+}$ has been performed to investigate the electron dynamics of the excited state via pump-probe scheme. Providing the linear pulse along with the molecular axis and ignoring the rotation of the molecule ion, the nuclear wave packets of two lowest electronic states (1s*σ*_*g*_ state and 2p*σ*_*u*_ state) can be obtained in the pump process by solving the two-state TDSE^24^, and then for each fixed *t*_*del*_, the harmonic emission from coherent state in the probe process can be obtained by solving the TDSE based on NBO approximation^25, 26^

1 $$i\frac{\partial }{\partial t}\psi (R,z;t)=H(R,z;t)\psi (R,z;t),$$

2 $$H(R,z;t)=-\frac{1}{M}\frac{{\partial }^{2}}{\partial {R}^{2}}-\frac{1}{2}\frac{{\partial }^{2}}{\partial {z}^{2}}+{V}_{c}(R,z)+kzE(t),$$

3 $${V}_{c}(R,z)=\frac{1}{R}-\frac{1}{\sqrt{{(z-R\mathrm{/2)}}^{2}+1}}-\frac{1}{\sqrt{{(z-R\mathrm{/2)}}^{2}-1}},$$

4 $$k=1+\frac{1}{2M+1},$$

where *M* is the mass of the nucleus, *R* is the internuclear distance and *z* is the electron coordinate (with respect to the nuclear center of mass).

The time-dependent wave function is advanced using the standard second-order split-operator method^26, 27^. In the numerical simulation, the converged numerical parameters are as follows: the grid ranges from 1.00 a.u. to 26.65 a.u. in *R* direction and from −202.50 a.u. to 202.50 a.u. in *z* direction, with a grid space of Δ*R* = 0.05 a.u. and Δ*z* = 0.15 a.u. With the exponential decay mask function employed in the work, the corresponding absorbing positions are 50 grids and 100 grids away from the boundaries in *R* direction and *z* direction, respectively. The corresponding time step is 0.2 a.u. The HHG spectrum can be obtained by Fourier transforming the time-dependent dipole acceleration $a(t)=\langle \psi (R,z;t)|-\frac{\partial {V}_{c}}{\partial z}+kE(t)|\psi (R,z;t)\rangle$ ^22, 28^, and the time-frequency distribution by means of the wavelet transform^23, 29–31^.

### Data availability

Data generated during this study is included in this article.
